# Supplementary material for: MetaQTL: a package of new computational methods for the meta-analysis of QTL mapping experiments
Source: BMC Bioinformatics. 2007 Feb 8;8:49. doi: 10.1186/1471-2105-8-49 (PMC1808479; doi:10.1186/1471-2105-8-49)
Supplement: Additional File 7 — MetaQTL Package : jar file and tutorial. This Zip archive contains both the MetaQTL JAR file and the files of the tutorial. [file 1471-2105-8-49-S7.zip › org.inra.metaqtl/doc/org/metaqtl/bio/entity/BioEntity.html]

BioEntity


|  |  |  |
| --- | --- | --- |
| **Overview** \| **Package** \| **Class** \| **Use** \| **Tree** \| **Deprecated** \| **Index** \| **Help** | |  |
| **PREV CLASS**   **NEXT CLASS** | **FRAMES**    **NO FRAMES**     **All Classes** |  |
| SUMMARY: NESTED \| FIELD \| CONSTR \| METHOD | DETAIL: FIELD \| CONSTR \| METHOD |  |


---


## org.metaqtl.bio.entity Class BioEntity

```
java.lang.Object
  org.metaqtl.bio.entity.BioEntity
```

**All Implemented Interfaces:**: IBioAdaptable, IBioEntity

**Direct Known Subclasses:**: BioEntityContainer, Ontology, OntologyTerm

---

``` public abstract class BioEntity extends java.lang.Object implements IBioEntity ```

Class Description Here

**Author:**
:   Jean-Baptiste Veyrieras

---

| **Field Summary** | |
| --- | --- |
| `protected  java.lang.String` | `name` |
| `protected  IBioEntity` | `parent` |
| `protected  java.util.Properties` | `properties` |


**Constructor Summary**

`BioEntity()`

`BioEntity(java.lang.String name,
IBioEntity parent)`


| **Method Summary** | |
| --- | --- |
| `java.lang.String` | `getName()`             Returns the name of the biological entity |
| `IBioEntity` | `getParent()`             Returns the entity that handles this entity. |
| `java.util.Properties` | `getProperties()`             Gets the properties of the entity. |
| `abstract  int` | `getType()`             There are 2 main class of entities. |
| `static IBioEntity` | `newBioEntity(int type)` |
| `void` | `setName(java.lang.String value)` |
| `void` | `setProperties(java.util.Properties defaultProp)`             Sets the properties of the entity. |

**Methods inherited from class java.lang.Object**

`clone, equals, finalize, getClass, hashCode, notify, notifyAll, toString, wait, wait, wait`

**Methods inherited from interface org.metaqtl.bio.IBioAdaptable**

`getBioAdapter`

**Field Detail**

### name

```
protected java.lang.String name
```

---


### parent

```
protected IBioEntity parent
```

---


### properties

```
protected java.util.Properties properties
```


**Constructor Detail**

### BioEntity

```
public BioEntity()
```

---


### BioEntity

```
public BioEntity(java.lang.String name,
                 IBioEntity parent)
```


**Method Detail**

### getName

```
public java.lang.String getName()
```

:   **Description copied from interface: `IBioEntity`**
:   Returns the name of the biological entity

    :   **Specified by:**: `getName` in interface `IBioEntity`

---


### setName

```
public void setName(java.lang.String value)
```

:   **Specified by:**: `setName` in interface `IBioEntity`

---


### getType

```
public abstract int getType()
```

:   **Description copied from interface: `IBioEntity`**
:   There are 2 main class of entities. The first one deals with population
    biological entity, i.e population itself and individuals. The second class
    is a representation of microscopic biological entity from genome container
    to alleles.

    :   **Specified by:**: `getType` in interface `IBioEntity`

---


### getParent

```
public IBioEntity getParent()
```

:   **Description copied from interface: `IBioEntity`**
:   Returns the entity that handles this entity.

    :   **Specified by:**: `getParent` in interface `IBioEntity`

---


### getProperties

```
public java.util.Properties getProperties()
```

:   **Description copied from interface: `IBioEntity`**
:   Gets the properties of the entity.

    :   **Specified by:**: `getProperties` in interface `IBioEntity`

---


### setProperties

```
public void setProperties(java.util.Properties defaultProp)
```

:   **Description copied from interface: `IBioEntity`**
:   Sets the properties of the entity.

    :   **Specified by:**: `setProperties` in interface `IBioEntity`

---


### newBioEntity

```
public static IBioEntity newBioEntity(int type)
```


---


|  |  |  |
| --- | --- | --- |
| **Overview** \| **Package** \| **Class** \| **Use** \| **Tree** \| **Deprecated** \| **Index** \| **Help** | |  |
| **PREV CLASS**   **NEXT CLASS** | **FRAMES**    **NO FRAMES**     **All Classes** |  |
| SUMMARY: NESTED \| FIELD \| CONSTR \| METHOD | DETAIL: FIELD \| CONSTR \| METHOD |  |


---
